# Normal variation in pelvic roll motion pattern during straight-line trot in hand in warmblood horses

**DOI:** 10.1038/s41598-023-44223-2

**Published:** 2023-10-10

**Authors:** A. Byström, A. M. Hardeman, M. T. Engell, J. H. Swagemakers, M. H. W. Koene, F. M. Serra-Bragança, M. Rhodin, E. Hernlund

**Affiliations:** 1 Department of Animal Environment and Health, Section of Ethology and Animal Welfare, Swedish University of Agricultural Sciences, Uppsala, Sweden; 2 Department of Clinical Sciences, Faculty of Veterinary Medicine, Utrecht University, Utrecht, The Netherlands; 3 Department of Companion Animal Clinical Sciences, Faculty of Veterinary Medicine, Equine Teaching Hospital, Norwegian University of Life Sciences, Oslo, Norway; 4 Tierklinik Lüesche GmbH, Lüesche, Germany; 5 Department of Anatomy, Physiology and Biochemistry, Swedish University of Agricultural Sciences, Uppsala, Sweden

**Keywords:** Biomechanics, Statistics

## Abstract

In horses, hip hike asymmetry, i.e. left–right difference in hip upwards movement during hind limb protraction in trot, is a crucial lameness sign. Vertical hip movements are complex, influenced by both pelvic roll and pelvic vertical motion. Veterinarians find it challenging to identify low-grade lameness, and knowledge of normal variation is a prerequisite for discerning abnormalities. This study, which included 100 clinically sound Warmblood horses, aimed to describe normal variation in pelvic roll stride patterns. Data were collected during straight-line trot in hand using optical motion capture. Stride-segmented pelvic roll data, normalised with respect to time (0–100% of the stride) and amplitude (± 0.5 of horse average stride range of motion), were modelled as a linear combination of sine and cosine curves. A sine curve with one period per stride and a cosine curve with three periods per stride explained the largest proportions of roll motion: model estimate 0.335 (p < 0.01) and 0.138 (p < 0.01), respectively. Using finite mixture models, the horses could be separated into three groups sharing common pelvic roll characteristics. In conclusion, pelvic roll motion in trot follows a similar basic pattern in most horses, yet there is significant individual variation in the relative prominence of the most characteristic features.

## Introduction

The horse is a highly valued animal in human society. Traditionally horses were used for transportation and agricultural labour, but today the horse is a recreational and sports partner. While humans greatly value the horse’s natural athletic capacity, lameness is unfortunately common in horses with an incidence of 20–50%^1–4^. Detection of lameness in horses is traditionally performed through visual assessment of the horse at steady-state locomotion, primarily in trot. Hind limb lameness is generally considered more difficult to detect than forelimb lameness, and studies on intra-rater agreement between veterinarians confirm that low-grade hind limb lameness is more challenging to detect consistently compared to forelimb lameness^5–7^. The most commonly described visual signs of hind limb lameness are asymmetric vertical motion of the pelvic midline/tubera sacrale, and left–right difference in the range of upwards movement of the hip (tuber coxae) during hind limb protraction, known as ’hip hike’^8^. Hip hike asymmetry is described to be more visually apparent, since vertical motion asymmetry of the pelvic midline tends to become magnified at the tubera coxarum due to simultaneous pelvic roll, i.e. axial rotation^9^. During the last two decades, measurement devices for objective gait analysis have gained increased use as an aid for detecting and quantifying motion asymmetries^10^. Scientific studies on methods for objective detection of gait abnormalities indicative of hind limb lameness have focused on measuring the movements underlying the above-described visual signs, i.e. parameters describing (a)symmetry of the vertical motion at the tuber sacrale and at the tubera coxarum (see e.g.^9,11^ for a list of commonly used parameters). Among these parameters, hip hike difference showed the largest change (15.4%) in response to diagnostic analgesia in hind limb lame horses^9^. However, hip hike asymmetries can be challenging to evaluate visually^12^. It has been shown that pelvic roll characteristics affect which aspect or phase of the hip motion that is most effective to focus on^12^, which implies that normal variation in pelvic roll can be a factor of importance during lameness assessments.

If both pelvic roll and pelvic vertical translation are known, then the vertical translation of the tubera coxarum can be calculated with good accuracy^13^. Further, if pelvic roll curve shape can be assumed to be largely similar in all horses, and to not be affected by lameness, then only pelvic vertical translation needs to be measured when performing objective gait analysis. Whether pelvic roll becomes asymmetric with lameness has been investigated in a modelling study using clinical data^13^. The results suggest that the left–right hip hike difference seen in hind limb lame horses encountered in the field can be explained from asymmetries in pelvic vertical motion alone. However, in that study the same pelvic roll stride curve, based on the data from four Thoroughbreds, was applied to all horses^13^. If there is substantial variation in pelvic roll pattern between horses, the importance of pelvic roll may therefore have been underestimated. From other studies, there are indications that the pelvic roll pattern varies between horses^14^ and there are brief, partly conflicting descriptions of changes in pelvic roll with lameness^15–18^. In summary, the value of measuring pelvic roll or tuber coxae vertical displacement is still an open question. For comparison, in human gait analysis quantifying pelvic rotation in all three planes is considered fundamental for understanding individual gait patterns^19,20^.

The main objective of the current study was to investigate whether horses show notable individual variation in pelvic roll pattern. When studying individual variation, it is preferable to avoid a priori selection of variables, because the range of features present across all individuals is unknown at the outset. In contrast to discrete point analysis, whole waveform analysis does not require calculation of time-discrete variables such as stride maximum, minimum or range of motion^21–23^, which eliminates the risk for investigator bias being introduced through variable selection. Within human biomechanics, techniques for whole waveform analysis originating from a variety of fields, including computer science, psychology, cognitive science, physics and engineering, have been applied and evaluated (e.g.^24,25^). Further, multivariate methods and various machine learning techniques have been applied to describe, for example, individual patterns^26,27^, gait variability^28^ and age-related changes^29^. Within equine biomechanics, whole waveform analysis have been used in a handful of studies. Fourier transformation^30,31^ and signal decomposition^32,33^ have been used to quantify vertical motion asymmetries. Statistical parametric mapping has been applied to analyse differences in fore- and hind limb ground reaction force curves between contra-lateral limbs^34,35^. Generalised additive model (GAM) analysis has been used to describe changes in rein tension throughout a training session^36^. Finally, various machine learning methods have been utilised, primarily to differentiate between sound and lame gait (see^37^ for an overview). However, whole waveform analysis is still scarcely used within equine biomechanics.

Thus, a further goal of the current study was to find methods for whole waveform analysis that are suitable for identifying and describing common patterns and individual variation in kinematic data. While machine-learning models are oftentimes effective in classifying data, for example, distinguishing between lame versus sound, they rarely provide information about the underlying basis for the model predictions^38^. In other words, the results are not straightforward to understand and interpret. Layer-wise relevance propagation has been proposed as general technique for explaining classifier decisions by decomposition, and has successfully been used to identify which time windows within the gait cycle that were most relevant for characterisation of individual gait patterns in humans^38^. However, this approach is quite complex. We wanted to find more simplistic methods that also produced biomechanically meaningful results. GAM is a subcategory of generalised linear models where a continuous response variable depends linearly on a number of unknown smooth functions of some predictor variables^39^. GAM is typically used to explore long-term trends^39^, but can be used to describe cycling variations if splines with a repetitive pattern are used, making it potentially suitable for analysis of stride data. Another interesting technique not yet utilised within biomechanics is finite mixture models, which is a type of cluster analysis popular for modelling unobserved heterogeneity^40^. Some of the benefits of finite mixture model analysis are that it does not require any prior assumptions about group characteristics, and that the suitable number of groups can be deduced from the data by assessing model fit and convergence^40^. The analyses used in the current study build on these two techniques, exploring their usefulness for describing stride patterns.

The aim of this study was to quantify and describe normal variation in pelvic roll during in-hand, straight-line trot in adult, clinically sound Warmblood sport horses, using a novel approach to analysing curve shape. As the whole waveform analyses used range-normalised data, a secondary aim was to investigate whether the horse’s age, the discipline the horse is trained in, or speed influenced pelvic rotation range of motion (ROM), as large such effects might confound the whole waveform analyses. It was hypothesised that horses show significant individual variation in pelvic roll pattern and that horses can be divided into subgroups with similar pelvic roll characteristics. It was further hypothesised that age, discipline and trotting speed would have a small but significant effect on pelvic rotation ROM.

## Materials and methods

### Horses

The study included data from non-lame Warmblood sport horses (age 2–16 years) from three different data collections: (1) eighty horses presented for pre-purchase exam (PPE)^41^; (2) eleven horses that participated in a study investigating normal variation in vertical motion symmetry^42^ and back ROM^43^; and (3) eight horses recruited for a study on pain expressions and associations to motion asymmetry. Further details including inclusion and exclusion criteria for each group can be found in Supplementary Table S1.

For groups 1 and 2, data collection took place at Tierklinik Lüesche, Germany, while for group 3 data were collected at Utrecht University equine clinic, the Netherlands. For studies 1 and 2 ethical permission was not required according to German law, given that they were non-invasive and did not subject the horses to any particular risks. For study 3 the study design and experimental protocol were approved by the Ethics Committee on the Care and Use of Experimental Animals at Utrecht University in compliance with Dutch legislation on animal experimentation (permission number AVD108002015307WP16, date of approval 18-08-2017). All horse owners provided written informed consent. All horses were assessed for lameness by an experienced veterinarian prior to data collection and deemed to be sound.

### Data collection

**Figure 1:**
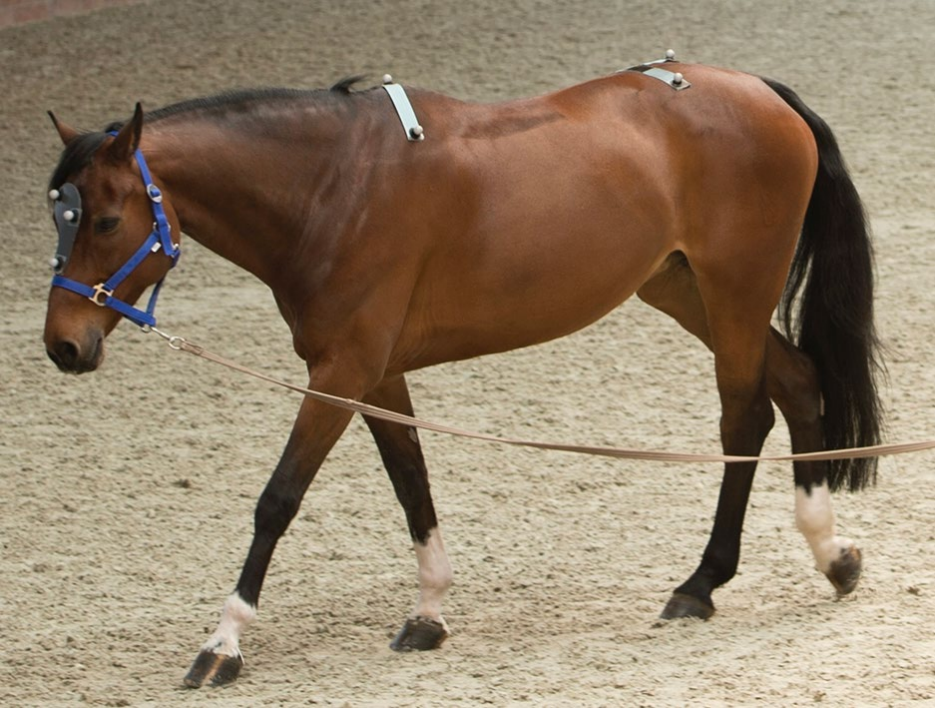
Marker placement. A T-shaped strip with one marker at each end was positioned with markers at the tuber sacrale and at the craniodorsal aspect of each tuber coxae. In addition, markers were placed on the withers (only the midline marker was used), and on the head (only the centre marker was used).

Kinematic data were collected using high-speed infrared cameras (Oqus 700+^a^, Qualisys AB, Sweden), sampling at 100 or 200 Hz depending on data collection site. Calibration was done before data collection according to the manufacturer’s instructions. Spherical reflective markers (25 mm) were placed midway between the two tubera sacrale and at the craniodorsal aspect of each tuber coxae using adhesive tape. For the horses in group 3 single separate markers were placed at these anatomical locations. For group 1 and group 2 a T-shaped, flexible rubber strip with one marker at each end was used (Fig. 1). Because the strip was available in three standardised sizes (pony, cob, full), the location of the tuber coxae markers varied slightly between horses. However, given that the pelvis is a rigid segment, this had a minimal influence on the rotation angles. Markers were also placed on the horse’s head (three markers attached to a flexible rubber sheet; only the central marker was used) and at the highest point of the withers (three markers attached to a band-shaped rubber strip; only the midline marker, placed over the spinous process of T5/T6, was used).

At their own preferred speed, horses were trotted 20 or 30 m back and forth on a straight line repeated twice. The surface consisted of sand and synthetic fibre at both data collection sites. Group 1 horses (presented for PPE) were trotted up by their owner/trainer, while the remaining horses were trotted up by a technician or research staff member. One trial per horse was recorded, however, if the horse showed irregular gait such as bucking or head tossing, the measurement was repeated.

### Kinematic data analysis

Kinematic raw data were recorded using the motion capture software for the camera system (QTM^a^ version: 2.14, build: 3180). Marker coordinate data were exported to Matlab (R2019b, Mathworks Inc., USA) and analysed using custom scripts.

Speed was calculated by differentiation of the horizontal coordinates (x and y) for the marker at the tubera sacrale. Pelvic roll, pitch and yaw were calculated as projection angles in the frontal, sagittal and dorsal planes, respectively. Pelvic roll was calculated using data from the tubera coxarum markers, and expressed relative to the horizontal. Pelvic roll was defined as zero when the tubera coxarum markers were level and positive if the right-side marker was lower than the left-side marker. Pelvic pitch was calculated using the marker at the tubera sacrale and the mean of the two tuber coxae markers, which was used as a virtual sagittal plane marker. Pelvic yaw was calculated using the two tuber coxae markers. Pelvic pitch and yaw were both expressed relative to a line between the withers marker and the tubera sacrale marker (calculated as a cross product with that line), to account for the horse’s orientation. Pelvic pitch was positive if the virtual sagittal plane marker was below the line between the withers and the tubera sacrale (corresponding to lumbosacral extension). Pelvic yaw was positive if the right tuber coxae marker was relatively further forward than the left tuber coxae marker (corresponding to right lateral flexion).

Time series data for the vertical displacement of the head and tubera sacrale markers, and for the pelvic rotations, were split into strides based on vertical maxima for the tubera sacrale marker (approximately corresponding to hind limb hoof-on). Pelvic roll was then used to determine left and right hind limb stance^14^, and the data were split into strides such that each stride started with left hind stance. Stride ROM was calculated for the three pelvic rotations, and for head and tubera sacrale vertical displacement. Single strides were excluded if stride duration or sacrum vertical ROM differed more than 20% from measurement median, or if head vertical ROM differed more than 40%. The stride-segmented data for pelvic roll were then time-normalised to 200 data points. A stride index ranging from 0 to 100% was then constructed as a relative time vector.

### Statistical analysis

All statistics were performed in R (version 3.6.2). In addition to the packages mentioned below, plyr, dplyr and reshape2 were used for data management, and ggplot2 for illustrations.

#### Group-level pelvic roll characteristics and presence of individual variation in curve shape

To analyse similarities and variation in roll curve shape between horses, an approach was taken that is similar in principle to GAM analysis. Pelvic roll data, time-normalised to 200 data points (0–100% of the stride) and amplitude-normalised to horse average range of motion (across all strides, ± 0.5 of ROM), were used as outcome variable. These data were modelled as a linear combination of sine and cosine curves with a successively increasing number of periods per stride, using a mixed model (R-package lmer). Each sine or cosine curve was included in the model as a function of data point index (stride percentage). For example, the term cos((index/100) * 2 * pi) represents a cosine curve with one period per stride (the model formula can be found in Supplementary Data S1, sheet ‘Explanations’). Both sine and cosine terms were included for each of the selected number of periods per stride to allow the phase (location of minima and maxima) to vary across the complete stride cycle. The range of periods per stride to evaluate in the model was based on visual assessment of the frequency content in the data (using fast Fourier transform in Matlab). It was decided to include sine and cosine curves with one, three, five, and seven periods per stride to represent symmetrical variations, and sine and cosine curves with two and four periods per stride to represent asymmetrical variations in the roll curves. Random terms included were horse, and sine and cosine curves with one period per stride nested within horse. The full model was backwards reduced. First it was confirmed that all random terms improved the Akaike information criterion (AIC) when tested upon the full model. Next, fixed effect terms with a p-value > 0.05 were removed, starting with the term with the largest p-value. Last, an anova comparing the final model to a model without the random slopes included was performed, in order to evaluate if the random slopes, which represent within-horse variation, contributed significantly to explaining the overall variation in the data. Normal distribution was confirmed from Q-Q-plots of model residuals and homoscedasticity was assessed by plotting fitted versus predicted values.

#### Identification of subgroups with similar pelvic roll characteristics

To investigate if the variation in roll curve shape between horses showed a homogeneous distribution or if there were groupings in the data, finite mixture models were used. The analysis was performed using the flexmix R-package^40^, selecting the driver FLXMRlmm. For this analysis, the data were reduced to one average curve per horse (consisting of 200 data points). This was necessary since nested random factors were not supported. In the model for all horses (described above) it was found that that a sine curve with one period per stride and a cosine curve with three periods per stride together described the most prominent features of the roll curve for all horses collectively. These two curves were therefore chosen as fixed effects, included as a function of data point index as described above. Random effect was horse. Number of cluster groups was set to one initially and then increased until the model converged with duplicate cluster groups (two or more groups with near identical estimates for all fixed effects). Unique models (without duplicate groups) were compared using AIC and visual assessment of rootograms (i.e. histograms for the square root of the count for each group) to find the model which fitted the data the best.

#### Influence of age, discipline and speed on pelvic ROM

Influence of age and discipline on pelvic range of motion was evaluated using mixed models (R-package lmer) with pelvic roll, pitch and yaw stride ROM as outcome variable. Models were made on stride-by-stride data. Fixed effects were age (continuous), discipline (categorical, dressage/show jumping/general purpose), the interaction between age and discipline, and speed (continuous). If the interaction was not significant (p > 0.05), models were re-run without the interaction. Horse was included as random effect. Models were also made including just speed as fixed effect, to specifically address the influence of speed. The purpose of latter models was mainly to assess speed as a possible cofounder to other analyses, where speed was not included. Normal distribution and homoscedasticity were checked as described for the curve shape model. Contrast p-values (two-tailed) were adjusted for multiple comparisons within each model using the Tukey method (R-package emmeans).

## Results

Three horses were excluded from the analysis due to incomplete motion capture data (poor tracking of relevant markers). The models for pelvic roll curve shape thus included 100 horses, of which 51 were dressage horses, 35 were show jumpers and 14 other disciplines/general purpose. Due to a lost document, age was unknown for two horses (both general purpose). The models for pelvic roll, pitch and yaw ROM therefore included 98 horses. The number of strides with data per horse was median 17 (range 8–25) for pelvic roll and median 16 (range 6–25) for pelvic pitch and yaw. Descriptive statistics can be found in Supplementary Table S2.

### Group-level common characteristics and individual variation in pelvic roll curve shape

**Figure 2:**
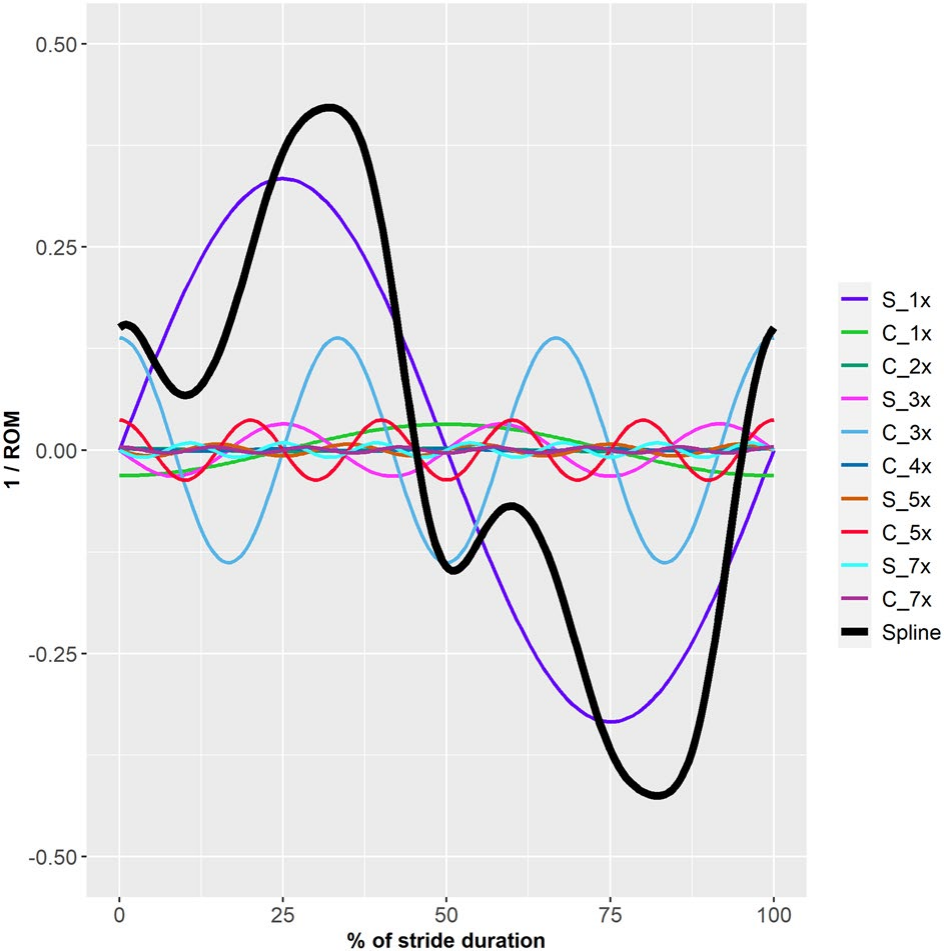
Graphical representation of model estimates from a mixed model for pelvic roll during trot (n = 100 horses) modelled as a linear combination of sine and cosine curves with 1–5 and 7 periods per stride. The coloured lines represent the sine (S) and cosine (C) curves that were significant at p < 0.05. Pelvic roll amplitude was normalised to ± 0.5 of horse average range of motion (ROM) and stride duration to 0–100%. The stride starts with left hind limb stance. Positive values indicate pelvic roll towards the horse’s right side. The amplitude of the coloured lines shows the size of the model estimate, i.e. the relative contribution (explained fraction) for each component. The thick black line shows the estimated curve from all significant terms combined, the amplitude indicates how well the model explained the data.

When the pelvic roll curve was modelled as a linear combination of sine and cosine curves, a sine curve with one period per stride and a cosine curve with three periods per stride showed the largest estimates (Fig. 2, Table 1). The inclusion of sine and cosine curves with one period per stride as random slopes significantly improved model fit (p < 0.0001).

**Table 1:** Model estimates from a mixed model for pelvic roll (n = 100 horses, one trial per horse) during trot modelled as a linear combination of sine and cosine curves with 1–5 and 7 periods per stride.

Curve	Period	Estimate	SE	P value
(Intercept)		− 0.056	0.030	0.069
Sin	1 x	0.335	0.010	< 0.001
Cos	1 x	− 0.026	0.011	0.022
Cos	2 x	0.002	0.001	< 0.001
Sin	3 x	− 0.032	0.001	< 0.001
Cos	3 x	0.138	0.001	< 0.001
Cos	4 x	0.001	0.001	0.013
Sin	5 x	− 0.007	0.001	< 0.001
Cos	5 x	0.037	0.001	< 0.001
Sin	7 x	− 0.009	0.001	< 0.001
Cos	7 x	0.004	0.001	< 0.001

Model estimates are shown for all sine (sin) and cosine (cos) curves that were significant at p < 0.05. Data were amplitude-normalised to ± 0.5 of horse average range of motion.

*SE* standard error.

### Identification of subgroups with similar pelvic roll characteristics

**Figure 3:**
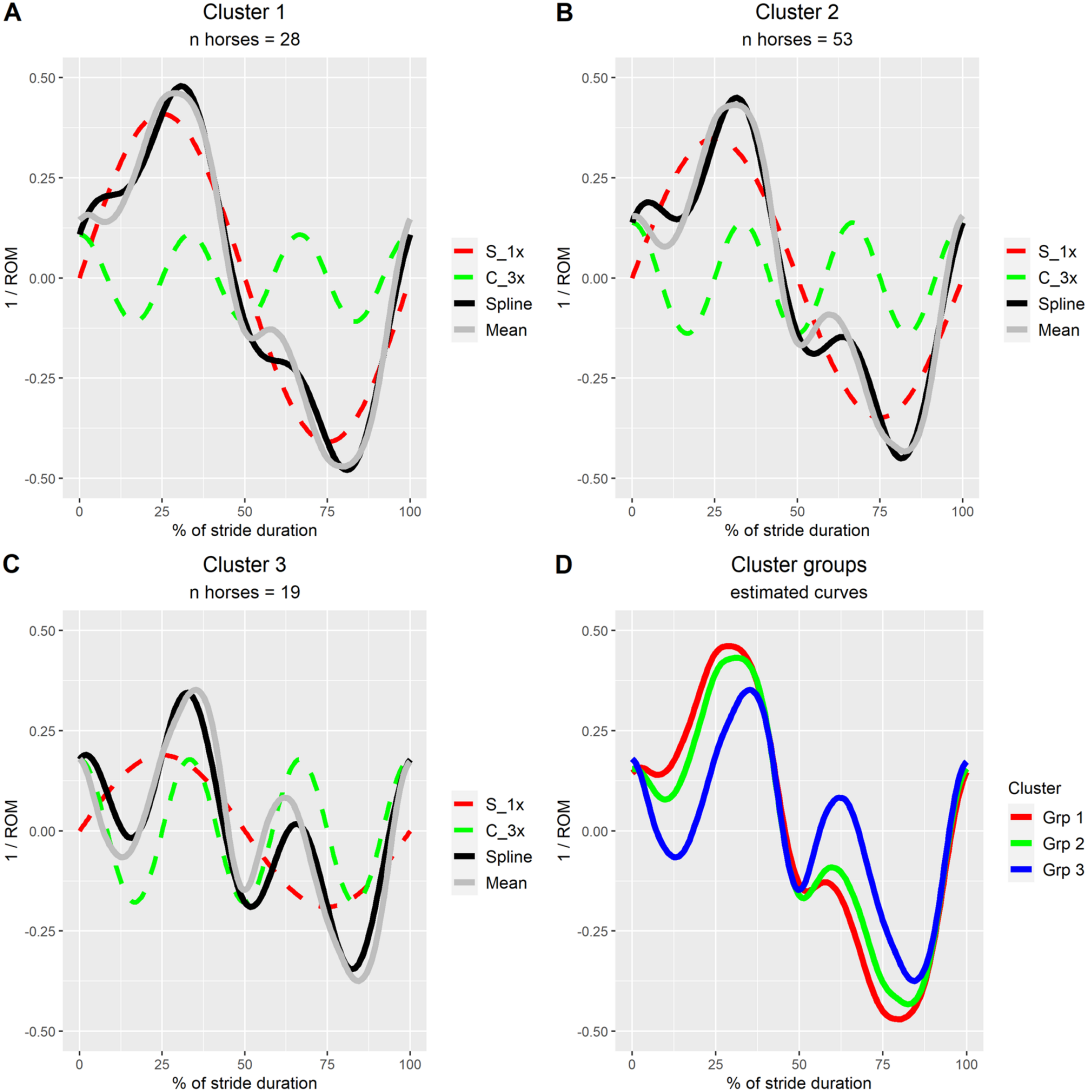
Illustration of model estimates from a finite fixture model for pelvic roll during trot (n = 100 horses) modelled as a linear combination of a sine curve with one period per stride (S_1x) and a cosine with three periods per stride (C_3x), selected based on the results displayed in Fig. 2. Pelvic roll amplitude was normalised to ± 0.5 of horse average range of motion (ROM) and stride duration to 0–100%, and data were averaged across available strides for each horse. Estimates for each cluster group (subgraphs **A**–**C**) are illustrated. In these, the amplitude of component curves (interrupted lines) indicates the relative contribution (explained fraction), the black line shows the compound curve estimate for each group (summation of sine and cosine curves), and the solid grey lines (Mean) show the group average curve (calculated from zero-centred and amplitude-normalised stride mean curves for each horse) for comparison. Graph (**D**) shows the compound curve estimate for all three cluster groups (black lines in **A**–**C**), coloured by group. The stride starts with left hind stance and positive values indicate pelvic roll towards the horse’s right side.

**Figure 4:**
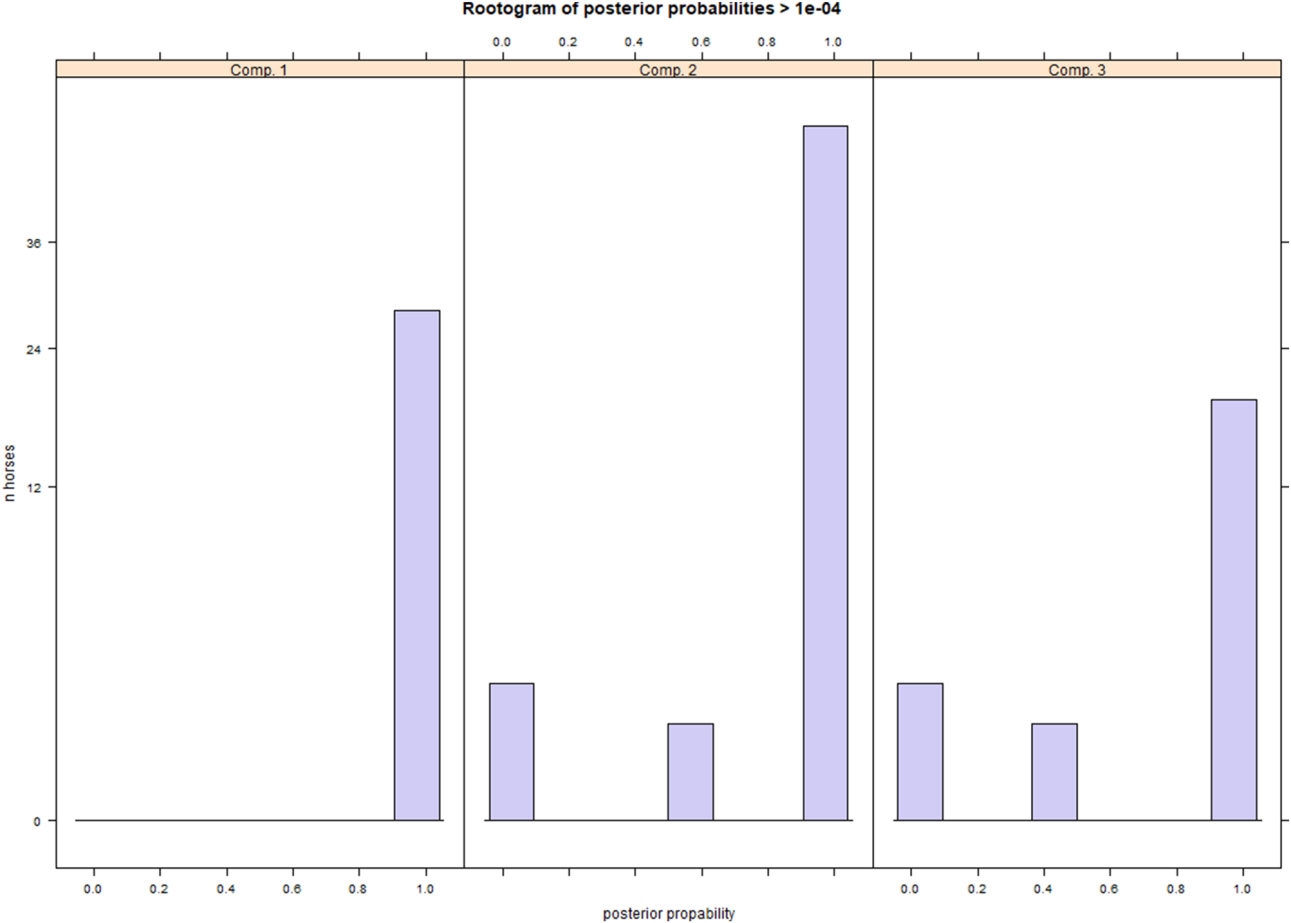
Rootograms for each cluster group (Comp 1–3) of the posterior probabilities from a finite fixture model with three cluster groups for pelvic roll during trot (n = 100 horses) modelled as a linear combination of a sine curve with one period per stride and a cosine curve with three periods per stride. The y-axis shows observation count on a square root scale. The x-axis scale indicates the probability of a horse to belong to the group in question. The theoretical total for each subgraph is the total n (100 horses), but observations with a probability ≤ 0.0001 are omitted. If the model fits the data perfectly, the expected appearance is for each group to have a single bar at 1.0 with a height equivalent to the group size (28 for group 1, 53 for group 2, 19 for group 3).

Further exploration of variation in pelvic roll features across the group of 100 horses, using finite mixture models, indicated that the horses could be separated into three cluster groups. Compared to models with one or two cluster groups, the model with three groups had the lowest AIC value. A model with four or five cluster groups converged with duplicate clusters (equivalent to non-conversion). Results for the model with three cluster groups are displayed graphically in Fig. 3. In Fig. 4, rootograms for the same model are displayed, which illustrate the separation of classes within each cluster. None of the three discipline groups were overrepresented in any of the three clusters. For example, 3 of 19 horses in cluster group 3 were general purpose. The same was true for the three data collection groups, indicating that data collection site and marker set-up (loose markers vs. fastened on a strip, see Fig. 1) had no prominent effects on curve shape. Model results for all finite mixture models, including AIC and rootograms, can be found in Supplementary File S2.

### Influence of age, discipline and speed on pelvic ROM

Age and discipline had few effects on pelvic range of motion. No significant interactions between age and discipline were found. Pelvic roll ROM had no significant association with age or discipline. Pelvic pitch ROM decreased significantly with age, -0.17 degrees per year (p = 0.012). Pelvic pitch ROM additionally differed significantly between disciplines. Post hoc tests showed a significant difference between dressage horses (estimated marginal mean [emm] 11.1 degrees) and the general purpose group (emm 9.0 degrees, p = 0.005). There was also a tendency for a difference between dressage horses and show jumpers (emm 10.1 degrees, p = 0.096). Pelvic roll ROM showed no significant correlation with speed. Pelvic pitch and yaw ROM decreased slightly with increasing speed, − 0.25 (SE 0.106) and − 0.21 (SE 0.079) degrees per 1 m/s, respectively. Full model printouts can be found in Supplementary File S1.

## Discussion

Using a novel approach to whole waveform analysis tailored for analysing stride-based data, the current study describes common pelvic roll features during straight-line trot in Warmblood sport horses, the type of horse most frequently used within the Olympic equestrian sports, as well as common variations in the typical pattern. The most prominent component was a sine curve with one period per stride, which appears to describe the global axial rotation of the pelvis related to the alternating protraction and retraction of hind limbs. The second most prominent feature was a local minimum/maximum that occurred during the impact phase (first half) of each hind limb stance, where pelvic roll momentarily reversed from rotation towards the limb in swing to rotation towards the limb in stance. This feature, represented by a cosine curve with three periods per stride, likely reflects some form of interaction between limb loading and simultaneous flexion and protraction of the contralateral limb. Both of these features are consistent with findings in previous studies on pelvic roll during trot^13,44^. Future studies may explore how these features relate to back and hind limb biomechanics, as well as to performance traits such as gait quality.

While a majority of horses displayed the same major pelvic roll features, the appearance and relative prominence of these varied among individuals. The inclusion of sine and cosine curves with one period per stride as random slopes nested in horse explained a significant proportion of the overall variation in curve shape within the study group, which suggests individual variation in the timing of the major pelvic roll minimum and maximum. Further investigation using finite fixture models revealed three common variations of the typical curve shape. These findings indicate that there is substantial individual variation in pelvic roll stride pattern among horses. The study only included Warmblood sport horses, meaning that all individuals were of similar height and body type. We expect that an even larger span of patterns would have been found if several different breeds had been represented, as other studies have found differences in motion patterns between breeds^45,46^. Interestingly, a previous study on methods for determining left and right hind limb stance phase from pelvic motion in trot found that inclusion of features representing pelvic roll components with both one and three periods per stride was crucial for achieving high classification accuracy (near 100% in the evaluated data sets)^14^. For comparison, 80–85% of strides were classified correctly if only the global minimum and maximum were considered^14^. The authors perceived that this was due to individual variation in the relative prominence of these two curve shape components.

Scientific studies have confirmed that low-grade hind limb lameness is challenging to detect consistently and accurately^5–7^. To aid veterinary students in learning to identify lameness, an online training tool has been developed, which uses 3D animations of horses with a varying degree of vertical motion asymmetry^47^. For hind limb lameness, students are first presented with a simplified pattern where the pelvis only moves vertically, before tasked with identifying vertical motion asymmetries in animations with more realistic pelvic motion. It was found that the students were significantly better at identifying hind limb lameness if pelvic roll was eliminated, both before and after training^47^. This supports that the complexity introduced by pelvic rotations contributes to the difficulty of identifying hind limb lameness. In these animations the same pelvic roll curve was applied in all cases. Individual variations in pelvic roll could potentially confuse visual signs of hind limb lameness even further. The influence of pelvic roll on the vertical motion of the hips depends both on pelvic roll amplitude and on its timing relative to minimum and maximum vertical displacement of the pelvis. Between the three subgroups identified in the current study, both the timing and the relative amplitude of the major pelvic roll peaks differ particularly when comparing cluster group 3 to the other two groups. This makes it plausible that the identified variations in pelvic roll affect the overall visual appearance of the horse’s pelvic motion. Further, these findings suggest that it is of value to measure both hip hike difference and vertical motion asymmetry of the pelvic midline when conducting an objective gait analysis. Clinicians should be aware of that individual differences in pelvic roll may result in varying discrepancies between vertical motion asymmetry measured at the sacrum and hip hike difference.

A major benefit of the methods for whole waveform analysis utilised in the current study is that the modelling approach is very flexible, since almost any cyclic pattern can be represented though selection of relevant sine and cosine components. If the dataset is sufficiently large it is also possible to test for interactions between different motion pattern components and other factors, e.g., associations between pelvic roll characteristics and breed, surface type or lameness. In other words, the methodology presented here will be a valuable addition to the biomechanist’s analytical toolbox, particularly for exploring previously unknown patterns and associations in biomechanical data. Since factors such as breed^45,46^ and level of training^48^ have a systematic influence on the horse’s motion pattern, knowledge of the expected pattern for a particular type of horse may be required to distinguish between normal variation and signs of lameness or other orthopaedic conditions. Studies have suggested that breed-specific reference values can be necessary to distinguish between normal and abnormal gait^46,49^. Increased knowledge about normal variations in pelvic motion could be helpful in order to identify abnormalities, such as motion pattern deviations associated with subtle or bilateral lameness, which is difficult to detect reliably with other methods. Future studies may explore how the data-driven approach for characterisation of curve shape used in the current study could be applied in a clinical context. This may include fingerprinting of individual horses as a baseline for continuous monitoring focused on lameness prevention or during rehabilitation, objective classification of normal motion patterns for different breeds, and identification of motion pattern components associated with various orthopaedic conditions.

While the mixed models analysis of curve shape used in the current study is novel, it builds on well-described and frequently used techniques. Mixed models are used for analysis of repeated measures data across a wide variety of disciplines within physical, biological and social sciences. Signal decomposition, including Fourier transformation, is commonly used for dimensionality reduction, e.g. in pre-processing of data for machine learning^14,37^. The current decomposition implementation, using mixed models, is similar to GAM, though slightly less complex since the main period is set to stride duration, rather than being a parameter in the model. Finite mixture model analysis is also a well-described technique^40^, only novel in this context. It allowed for separation of the horses into subgroups with similar patterns using a subset of the explanatory variables used in the mixed models, achieving continuity with the mixed models analysis. The identified subgroup patterns are useful for illustrating and better understanding of what variations in pelvic roll pattern that are common in Warmblood sport horses. In summary, our results support the usefulness of whole waveform analysis for describing individual patterns in biomechanical variables, in accordance with conclusions from previous studies on human gait^26,27^.

Pelvic rotation range of motion was analysed to complement the whole waveform analyses because the latter used range-normalised data (effectively ignoring ROM). This revealed that both discipline and age influenced pelvic pitch ROM, but not roll or yaw ROM. Dressage horses had a greater pitch ROM than general-purpose horses, and there was a tendency for the same difference compared to show jumpers. This might reflect that dressage horses commonly have more elastic and expressive movements. Older horses showed a slight decrease in pitch ROM, possibly due to increased stiffness with age. There was a slight decrease in pitch and yaw ROM with increasing speed, which may reflect increased back stabilisation at higher speeds, as was seen in a previous study^50^. However, this effect was small, and we have no reason to believe that speed variations between horses or within a measurement have confounded the whole waveform analyses, where speed was not included as a covariate. It should be noted that this study was not designed to investigate the effect of speed on pelvic rotations. The speed range within horse was small, mainly representing minor accelerations and decelerations of the horse during the run. Different results for the speed effects may have been found if steady-state locomotion across a more extensive range of speeds had been recorded.

The cluster groups determined through finite fixture model analysis are not the only possible representation of the variability in the current dataset. Different groups may have been found if different independent variables had been included in the finite mixture models. For example, including both sine and cosine curves with one period per stride resulted in a slightly different solution. It can also be appreciated from Fig. 3. that due to the simplified model formula, the finite mixture models were not able to represent all features of the pelvic roll pattern displayed by each cluster group, even though the major characteristics were captured. Further, it should be noted that﻿ some horses fitted their allocated subgroup less well (posterior probability < 1.0), which means that there was some individual variation also within each subgroup. The primary purpose of this analysis was not to completely describe all variations in the current dataset, rather to study how the main pelvic roll features identified at group level varied across individuals. Due to implementation limitations in the software package used for the finite mixture models, it was necessary to limit the set of curve shapes included; it was not possible to use the same module formula as in the mixed models. With future development of the flexmix R-package, it may become possible to analyse more complex models.

## Conclusions

Pelvic roll motion in straight-line trot followed a common basic pattern in the studied group of Warmblood riding horses. The two most prominent components were global axial rotation of the pelvis in conjunction with the alternating protraction and retraction of hind limbs, and a local minimum/maximum that occurred during the first half of each hind limb stance, which is in accordance with previous descriptions in the literature. However, there was substantial individual variation in the characteristics of this pattern. Using a data-driven approach, we could separate the horses into three distinct subgroups with different relative prominence of the two most prominent components. Due to the inherent relationship between pelvic roll and the vertical displacement of the hips, this individual variation may have an impact on both the visual impression and objective measurements obtained during lameness assessments. The simplistic yet powerful methods used for evaluation of curve shape exhibits exciting future potential for objectively classifying motion patterns specific to a breed, an individual, or various orthopaedic conditions.

### Supplementary Information

Supplementary Information 1.

Supplementary Information 2.

Supplementary Information 3.

Supplementary Table S1.

Supplementary Table S2.

## Data Availability

The data used for statistical analysis can be found in Supplementary Data S1.
