# Supplementary material for: Normal variation in pelvic roll motion pattern during straight-line trot in hand in warmblood horses
Source: Sci Rep. 2023 Oct 10;13:17117. doi: 10.1038/s41598-023-44223-2 (PMC10564842; doi:10.1038/s41598-023-44223-2)
Supplement: Supplementary file 4 — Supplementary Table S1. [file 41598_2023_44223_MOESM4_ESM.pdf]

Supplementary Table S1. Detailed description of the three included groups of horses.

	Group 1	Group 2	Group 3
Number of horses	80	11	8
Horse age	2-16 years (mean 7.2)	5-15 years	7.7 (SD 3.1)
Withers height	76 horses >1.48 m<br>4 ponies >1.45 m	>1.48 m	>1.48 m
Inclusion criteria	Presented for pre-purchase exam during the study period (May 2018 until December 2019).	Currently in ridden work.<br>Stabled in the vicinity of the data collection site (Tierklinik Lüesche, Germany)	Perceived as sound and healthy.<br>Eligible for participation in an experimental lameness study.
Exclusion criteria	Client or veterinarian did not want an objective motion analysis.<br>Significant veterinary concerns found during orthopaedic examination.	Clinical signs of lameness (grade >1/5 in trot) or signs of back dysfunction.	Clinical signs of lameness (grade >1/5 in trot).
