# Supplementary material for: Normal variation in pelvic roll motion pattern during straight-line trot in hand in warmblood horses
Source: Sci Rep. 2023 Oct 10;13:17117. doi: 10.1038/s41598-023-44223-2 (PMC10564842; doi:10.1038/s41598-023-44223-2)
Supplement: Supplementary file 5 — Supplementary Table S2. [file 41598_2023_44223_MOESM5_ESM.pdf]

Supplementary Table S2. Descriptive statistics for horse age and pelvic roll, pitch and yaw range of motion (ROM) during trot and number of strides per horse (n = 100 horses, one trial per horse) for the pelvic ROM variables. Age was unknown for two horses.

Variable	n horses	mean	SD	median	min	max
age	98	7.29	3.08	7	2	16
stride speed	100	3.56	0.37	3.56	2.67	4.44
Pelvis roll ROM	100	8.45	2.03	8.56	4.18	15.06
Pelvis pitch ROM	100	10.44	2.16	10.26	4.96	17.84
Pelvis yaw ROM	100	5.03	1.14	5.03	1.99	7.45
n Pelvis roll ROM	100	16.51	4	17	8	25
n Pelvis pitch ROM	100	16.02	4.27	16	6	25
n Pelvis yaw ROM	100	16.02	4.27	16	6	25

Variables prefixed with 'n' is the number strides per horse with data for the corresponding ROM variable
